# Association Between Sensory Loss and Falls Among Middle-Aged and Older Chinese Population: Cross-Sectional and Longitudinal Analyses

**DOI:** 10.3389/fmed.2021.810159

**Published:** 2022-01-11

**Authors:** Yifan Zhou, Yanping Hu, Jianfeng Luo, Yinwen Li, Haiyun Liu, Xiaodong Sun, Minwen Zhou

**Affiliations:** ^1^Department of Ophthalmology, Putuo People's Hospital, Tongji University, Shanghai, China; ^2^Shanghai Putuo Center for Disease Control and Prevention, Shanghai, China; ^3^School of Public Health, Fudan University, Shanghai, China; ^4^Department of Ophthalmology, Shanghai General Hospital (Shanghai First People's Hospital), School of Medicine, Shanghai Jiao Tong University, Shanghai, China; ^5^National Clinical Research Center for Eye Diseases, Shanghai, China; ^6^Shanghai Key Laboratory of Fundus Diseases, Shanghai, China

**Keywords:** hearing loss, vision loss, dual sensory loss, falls, CHARLS

## Abstract

**Introduction:** Previous studies have suggested that sensory loss is linked to falls. However, most of these studies were cross-sectional designed, focused on single sensory loss, and were conducted in developed countries with mixed results. The current study aims to investigate the longitudinal relationship between hearing loss (HL), vision loss (VL) and dual sensory loss (DSL) with falls among middle-aged and older Chinese population over 7 years.

**Methods:** The data was obtained from the China Health and Retirement Longitudinal Survey (CHARLS). In total, 7,623 Chinese older adults aged over 45 were included at baseline 2011 in this study. Self-reported falls and HL/VL/DSL were accepted. Other confounding variables included age, sex, BMI, educational level, marital status, various physical disorders and lifestyles. The impact of baseline sensory status on baseline prevalence of falls and incident falls over 7 years were assessed using logistic regression analyses. A logistic mixed model was used to assess the association between time-varying sensory loss with incident falls over 7 years after adjusted with multi-confounding factors.

**Results:** Single and dual sensory loss groups had significantly higher prevalence of falls compared to no sensory loss (NSL) group (DSL: 22.4%, HL: 17.4%, VL: 15.7%, NSL: 12.3%). Baseline HL (OR: 1.503, 95% CI: 1.240–1.820), VL (OR: 1.330, 95% CI: 1.075–1.646) and DSL (OR: 2.061, 95% CI: 1.768–2.404) were significantly associated with prevalence of falls. For longitudinal observation over 7 years, baseline HL/DSL and persistence of all types of sensory loss were associated with incidence of falls. Time-varying HL (OR: 1.203, 95% CI: 1.070–1.354) and DSL (OR: 1.479, 95% CI: 1.343–1.629) were associated with incident falls after adjusted with multi-confounders, while VL was not.

**Conclusion:** HL and DSL are significantly associated with both onset and increased incidence of falls over 7 year's observation in middle-aged and elderly Chinese population. Persistence or amelioration of sensory loss status could exert divergent influences on incidence of falls, which should be considered in the development of falls-prevention public health policies for aging population.

## Introduction

Falls and fall-induced injuries are leading causes of morbidity and mortality among older people (1). Falls can cause moderate to severe events, such as bone fractures, head trauma, or even increased risk of early death (1). Among elderly people, falls are the leading cause of death due to injury. The frequency of falls increases with aging. Approximately, 28–35% of people all over the world aged over 65 fall every year, and this number increases to 32–42% for those who aged over 70 (2). With the rapid growth of the world's older population, falls has become a major concern of the public health problems worldwide.

As the world's most populous country, China has accelerated aging population with increasing average life expectancy. It is estimated that the number of Chinese people aged 80 years or older will quadruple over the next two decades (3, 4). Until now, according to WHO reports, the annual prevalence of falls among older Chinese population has reached 6.5 to 30.6% (3). Thus, falls, fall-induced injuries and related events in older Chinese population are of great significance. To date, numerous researchers have explored the incidence, risk factors and socio-economic burden of fall and related injuries in Chinese population (4). As for risk factors, some have mentioned the association of sensory loss with falls (5–8).

Sensory loss, consisting of hearing loss (HL), vision loss (VL), and dual sensory loss (DSL, co-occurrence of vision and hearing loss), is one of the most common problems experienced by older people. Although consensus has yet to be reached, the relationship between sensory loss and falls in older population had aroused great concern in both developed and developing countries (6, 9–13). Among older Chinese, the prevalence of self-reported HL, VL, and DSL is relatively higher than the prevalence reported in many developed countries (14). Due to traditional attitudes regarding sensory loss as a normal part of aging life, older Chinese people are likely to ignore problems related to sensory loss, which may further contribute to the higher prevalence of sensory loss and incidence of related problems in our population (14).

Very recently, a small number of cross-sectional studies have reported the potential relationship between vision impairment and falls/fall-related injuries among Chinese population (5, 6, 8). Therefore, longitudinal study is needed. However, researches on hearing loss and falls have yield mixed results (6, 10, 15, 16). Also, the impact of Dual Sensory Loss (DSL) on falls has been barely explored before in our population as well. Thus, allowing for the specific cultural background, attitudes toward sensory loss, and public health system in mainland China, it is necessary to investigate the longitudinal correlation between sensory loss (vision, hearing or both) and falls among older Chinese population.

The China Health and Retirement Longitudinal Study (CHARLS) is the first nationally representative survey of the health status and well-being in middle-aged and older population in China, which provides high-quality longitudinal data of massive amounts of personal health-related information including sensory loss and self-reported falls. The purpose of this study is to verify single/dual sensory loss as risk factors of falls among older Chinese population according to cross-sectional study and longitudinal observation spanning 7 year of follow-up.

## Methods

### Participants and Public Involvement

**Figure 1:**
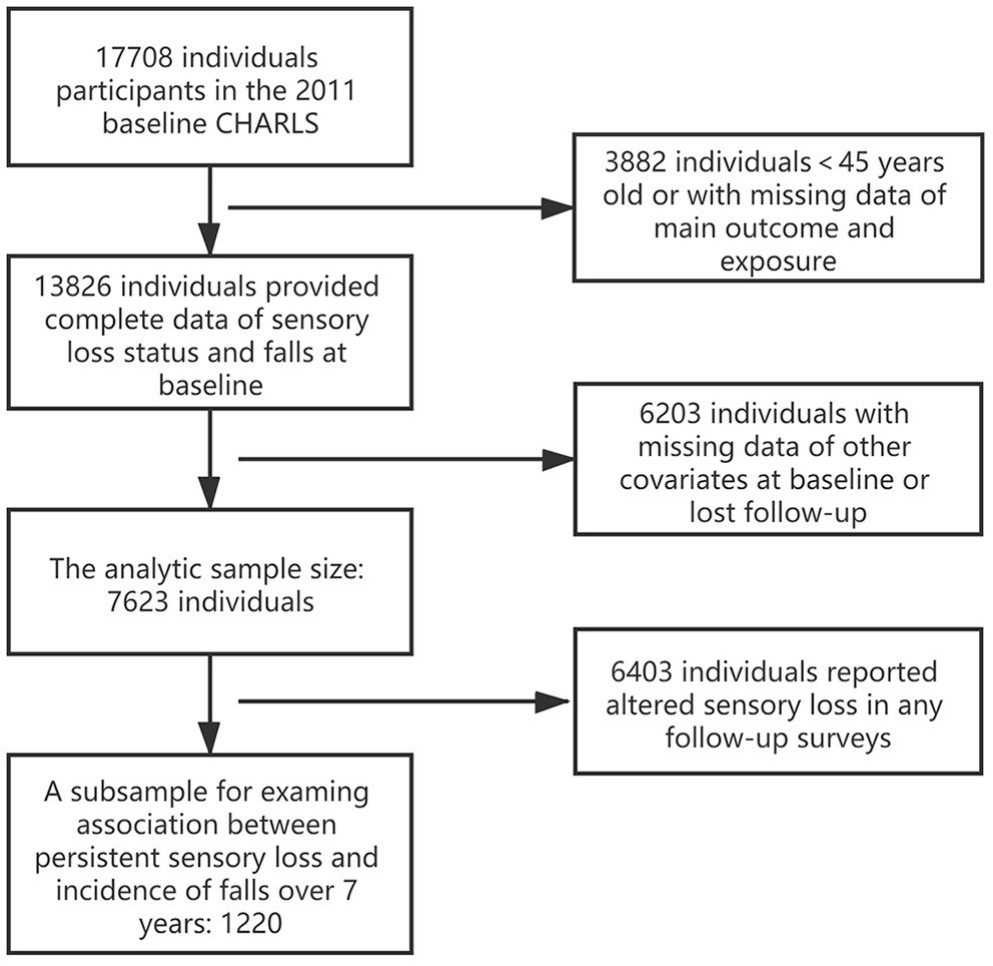
Sample reduction from the 2011 baseline sample of China Health and Retirement Longitudinal Survey (CHARLS) to current analytic sample.

We obtained data from the China Health and Retirement Longitudinal Study (CHARLS), the first nationally representative longitudinal survey sampling residents (middle-aged and older adults, over 45 years old) from 450 villages/neighborhoods, 150 counties across 28 provinces in China. With response rate over 80%, CHARLS provides the most up-to-date longitudinal data sets for studying the health status and well-being of middle-aged and older population in China. There were 17,708 participants interviewed in the 2011 baseline (Wave 1). According to the purpose of the current study, participants with missing data of any variables at 2011 baseline and lost follow-up in falls and sensory loss were excluded, which led to a final sample size of 7,623 (Figure 1).

### Measures

#### Outcome

The main outcome in this study is falls, which was determined by the question “Have you fallen down in the last 2 years?” Possible answers included “yes” or “no.” We therefore treated the outcome variable as binary. In each follow-up survey, the participants were asked “Have you fallen down since the last interview?” Baseline assessment of falls was used for cross-sectional study and incidence of falls reported during 3 waves of follow-up were used for longitudinal analysis.

#### Exposure

The main exposure in this present study are self-reported vision loss and hearing loss. In CHARLS, vision loss (VL) included distal vision impairment and near vision impairment. Distal vision impairment was assessed by asking the participants whether their eyesight was excellent, very good, good, fair, or poor when seeing things at a distance. Reporting of fair or poor eyesight was classified as distal vision impairment. Near vision impairment was assessed by asking participants whether their eyesight was excellent, very good, good, fair, or poor when seeing things up close. Reporting of fair or poor eyesight was classified as near vision impairment. To assess hearing loss (HL), the question was: “Is your hearing excellent, very good, good, fair, poor (with a hearing aid if you normally use it and without if you normally don't).” A response to this question stating fair or poor hearing was classified as HL. Such categorization of sensory loss assessment was used in previous studies (17–19). DSL referred to participants with both VL and HL.

In realized that the status of sensory loss could alter during 7 years of observation, we thought that it might be less prudent to only consider the baseline sensory loss status and its association with falls. Persistent exposure to specific sensory loss and altered sensory loss statuses during follow-up should be taken into account in longitudinal study as well. Thus, to assess the impact of persistent exposure condition of different types of sensory loss status on incidence of falls in our participants, the answer to vision/hearing status at each follow-up time point should be the same (e.g., participants with cataract-caused vision impairment and visual improvement after cataract surgery would probably report different vision statuses at different timepoints. Such participants would be regarded as break down of persistent vision loss status and excluded). On the other hand, participants without sensory loss at baseline could develop sensory impairment during follow-up for 7 years and vice versa. Thus, we further treated sensory loss statuses of our participants as time-varying variable to tolerate alterations in surveys at different time points over 7 years. The impact of time-varying sensory loss on incidence of falls appeared during follow-up was also assessed.

### Other Variates

#### Socio-Demographic Characteristics

Gender was a binary variable: male and female. Age was treated as a continuous variable. Marital status indicated whether the respondent lived alone or got accompanied. Participants who were separated, divorced, widowed or never married were coded as “living alone” group, while those who were married or partnered were coded as “living with partner” group. Educational attainment was used to represent social economic status, which could probably affect people's access to health services and other social and economic resources. Educational status was categorized into 5 groups: illiterate, less than elementary school, elementary school, middle school and high school or above as previously reported (20, 21).

#### Lifestyle

The lifestyle variables included smoking status, drinking status, and physical activities status. Smoking status was categorized as current/former smoker or never smoked. Drinking was a 3-category variable indicating the frequency of drinking: none, less than once a month and more than once a month. Physical activities status was categorized as taking vigorous activity, moderate activity, light activity, or insufficient activity.

#### Physical Disorders

In CHARLS, most health status and physical disorders were assessed according to self-reports. Only a few diseases could be defined at a relatively precise level based on both self-reported medical history and reference definition like blood test results and physical examinations. Thus, we took only seven main physical disorders into account in the present study: hypertension (22, 23), diabetes (24, 25), dyslipidemia (22, 26), kidney diseases (27), emotional disorders, memory-related diseases and stroke (28). The criteria of identifications of physical disorders used in the current study was also adopted by numerous researchers using CHARLS datasets.

### Statistical Analysis

Statistical analyses were performed using SAS, version 9.4 (SAS Institute, Cary, NC, US). In this study, the primary exposure of interest was sensory loss status (HL/VL/DSL), while the other independent variables served as control variables and were reported as means ± SD or numbers (%). Baseline characteristics were compared between participants with different sensory loss statuses (4 groups) using the Chi-square test, Cochran-Mantel-Haenszel (CMH) test or analysis of variance depending on the data type and distribution. Logistic regression analyses were conducted to assess the associations between prevalence/incidence of falls and baseline/persistent sensory loss. While the longitudinal associations between time-varying sensory loss and incident falls were examined using mixed logistic models. Mixed logistic models took into account within-subject correlation of time-varying sensory loss and fall over 7 years of follow-up (3 waves). Multi-confounders including socio-demographic factors, lifestyles and physical disorders were adjusted in logistic models.

## Results

In total, 7,623 Chinese older adults aged over 45 at baseline 2011 were deemed eligible for the current study (Figure 1). Socio-demographic characteristics, physical conditions and lifestyles of the study sample were grouped by sensory status and presented in Table 1. The number of participants at baseline was 7,263 in the current study. For each group, the sample size was 2,136 (NSL), 1,320 (HL), 1,004 (VL), 3,163 (DSL). The DSL group had the highest prevalence of falls (22.4%, *n* = 3,163). Participants with single sensory loss had relatively higher prevalence of falls than those who without sensory loss (HL: 17.4%, VL: 15.7%, NSL: 12.3%, *p* < 0.001).

**Table 1:** Baseline characteristics of participants by sensory loss.

**Variables**	**No sensory loss**	**Hearing loss**	**Vision loss**	**Dual sensory loss**	***P*-value**
	**(*N* = 2,136)**	**(*N* = 1,320)**	**(*N* = 1,004)**	**(*N* = 3,163)**	
**Falls**					<0.01
Yes	263 (12.3%)	230 (17.4%)	158 (15.7%)	710 (22.4%)	
No	1,873 (87.7%)	1,090 (82.6%)	846 (84.3%)	2,453 (77.6%)	
**Gender**					<0.01
Male	1,071 (50.1%)	711 (53.9%)	432 (43.0%)	1,435 (45.4%)	
Female	1,065 (49.9%)	609 (46.1%)	572 (57.0%)	1,728 (54.6%)	
**Age**					<0.01
	59.80 ± 9.20	63.17 ± 9.73	61.75 ± 9.17	64.09 ± 9.30	
**BMI**					<0.01
	23.51 ± 4.10	23.20 ± 4.03	23.35 ± 3.81	23.10 ± 3.81	
**Marital status**					<0.01
Yes	1,762 (82.5%)	1,052 (79.7%)	841 (83.8%)	2,495 (78.9%)	
No	374 (17.5%)	268 (20.3%)	163 (16.2%)	668 (21.1%)	
**Educational level**					<0.01
Illiterate	530 (24.8%)	383 (29.0%)	315 (31.4%)	1,127 (35.6%)	
Less than elementary school	371 (17.4%)	275 (20.8%)	202 (20.1%)	667 (21.1%)	
Elementary school	477 (22.3%)	304 (23.0%)	221 (22.0%)	729 (23.0%)	
Middle school	459 (21.5%)	233 (17.7%)	174 (17.3%)	460 (14.5%)	
High school or above	299 (14.0%)	125 (9.5%)	92 (9.2%)	180 (5.7%)	
**Smoking status**					<0.01
Yes	860 (40.3%)	590 (44.7%)	359 (35.8%)	1,233 (39.0%)	
No	1,276 (59.7%)	730 (55.3%)	645 (64.2%)	1,930 (61.0%)	
**Drinking status**					<0.01
Never	553 (25.9%)	351 (26.6%)	237 (23.6%)	740 (23.4%)	
Drink more than once a month	175 (8.2%)	106 (8.0%)	63 (6.3%)	228 (7.2%)	
Drink but less than once a month	1,408 (65.9%)	863 (65.4%)	704 (70.1%)	2,195 (69.4%)	
**Physical activity**					<0.01
Vigorous	760 (35.6%)	393 (29.8%)	352 (35.1%)	871 (27.5%)	
Moderate	583 (27.3%)	365 (27.7%)	293 (29.2%)	837 (26.5%)	
Light	454 (21.3%)	293 (22.2%)	212 (21.1%)	751 (23.7%)	
Insufficient	339 (15.9%)	269 (20.4%)	147 (14.6%)	704 (22.3%)	
**Diabetes**					<0.01
Yes	244 (11.4%)	180 (13.6%)	166 (16.5%)	462 (14.6%)	
No	1,892 (88.6%)	1,140 (86.4%)	838 (83.5%)	2,701 (85.4%)	
**Hypertension**					<0.01
Yes	953 (44.6%)	648 (49.1%)	467 (46.5%)	1,576 (49.8%)	
No	1,183 (55.4%)	672 (50.9%)	537 (53.5%)	1,587 (50.2%)	
**Dyslipidemia**					0.981
Yes	707 (33.1%)	431 (32.7%)	335 (33.4%)	1,038 (32.8%)	
No	1,429 (66.9%)	889 (67.3%)	669 (66.6%)	2,125 (67.2%)	
**Kidney diseases**					<0.01
Yes	322 (15.1%)	291 (22.0%)	183 (18.2%)	748 (23.6%)	
No	1,814 (84.9%)	1,029 (78.0%)	821 (81.8%)	2,415 (76.4%)	
**Emotional problems**					0.07
Yes	15 (0.7%)	14 (1.1%)	13 (1.3%)	47 (1.5%)	
No	2,121 (99.3%)	1,306 (98.9%)	991 (98.7%)	3,116 (98.5%)	
**Memory-related diseases**					<0.01
Yes	12 (0.6%)	17 (1.3%)	15 (1.5%)	72 (2.3%)	
No	2,124 (99.4%)	1,303 (98.7%)	989 (98.5%)	3,091 (97.7%)	
**Stroke**					0.02
Yes	35 (1.6%)	36 (2.7%)	30 (3.0%)	92 (2.9%)	
No	2,101 (98.4%)	1,284 (97.3%)	974 (97.0%)	3,071 (97.1%)	

The univariate logistic regression analysis indicated the potential associated factors of fall in our sample at baseline 2011. Sensory loss including VL, HL, and DSL, along with other factors including gender, age, marital status, educational level, smoking status, physical activities status, diabetes, hypertension, kidney diseases, emotional problems, memory-related diseases and stroke were all found to be significantly associated with fall (all *p* < 0.05, Table 2). Compared to single sensory loss, DSL had a higher odds ratio, which means a potentially greater impact on prevalence of falls (OR-DSL: 2.061, OR-HL: 1.503, OR-VL: 1.330).

**Table 2:** Univariate logistic regression model to describe the correlation between univariates and prevalence of falls at baseline.

**Variables**	**OR (95% CI)**	***P*-value**
**Main exposure**
No sensory loss	Reference	
Hearing loss	1.503 (1.240,1.821)	<0.01
Vision loss	1.330 (1.075,1.646)	<0.01
Dual sensory loss	2.061 (1.768,2.404)	<0.01
**Gender**
Female	Reference	
Male	0.734 (0.652,0.827)	<0.01
Age	1.021 (1.015,1.027)	<0.01
BMI	1.003 (0.988,1.018)	0.72
Marital status		<0.01
Living with partner	Reference	
Living alone	1.220 (1.057,1.408)	
**Educational level**
Illiterate	Reference	
Less than elementary school	0.935 (0.797,1.096)	0.41
Elementary school	0.713 (0.607,0.838)	<0.01
Middle school	0.605 (0.504,0.727)	<0.01
High school or above	0.462 (0.358,0.597)	<0.01
Smoking	1.146 (1.015,1.293)	0.03
**Drinking status**
Never	Reference	
Drink more than once a month	1.109 (0.968,1.271)	0.14
Drink but less than once a month	1.178 (0.947,1.464)	0.14
**Physical activity**
Insufficient	Reference	
Vigorous	0.760 (0.643,0.899)	<0.01
Moderate	0.754 (0.635,0.896)	<0.01
Light	0.960 (0.806,1.142)	0.64
Diabetes	1.391 (1.187,1.630)	<0.01
Hypertension	1.194 (1.061,1.342)	<0.01
Dyslipidemia	1.007 (0.889,1.141)	0.91
Kidney diseases	1.278 (1.112,1.470)	<0.01
Emotional problems	2.621 (1.693,4.058)	<0.01
Memory-related diseases	1.547 (1.012,2.364)	0.04
Stroke	1.587 (1.141,2.206)	<0.01

The results of the univariate logistic regression indicated probabilities that certain covariables that could confound the relationship between sensory loss and falls in multivariate regression models. Table 3 showed the impact of baseline sensory status on prevalence of falls at baseline and incidence of falls over 7 years according to adjusted multivariable logistic models. At baseline, HL and DSL remained significantly correlated with falls in all 4 Models. VL was found to be significantly correlated with falls in Model 1&2&3, however, after being adjusted with various physical disorders, such correlation become less significant (*p* = 0.08). Similarly, baseline HL and DSL were found to have significant correlation and prediction for higher incidence of falls over 7 year follow-up longitudinal observation after being adjusted with multiple confounding factors, but it was not the case of VL (Table 3).

**Table 3:** Multivariate logistic regression models for the associations between baseline sensory loss, baseline prevalence of falls and incident falls over 7 years.

	**Baseline sensory loss and baseline prevalence of falls**							
**Variables**	**Model 1**		**Model 2**		**Model 3**		**Model 4**	
	**OR**	**95%CI**	**OR**	**95%CI**	**OR**	**95%CI**	**OR**	**95%CI**
**No sensory loss**	**Reference**		**Reference**		**Reference**		**Reference**	
Hearing loss	1.503^***^	(1.240, 1.821)	1.440^***^	(1.186, 1.748)	1.417^***^	(1.166, 1.721)	1.394^***^	(1.147, 1.694)
Vision loss	1.330^**^	(1.075, 1.646)	1.260^*^	(1.017, 1.562)	1.246	(1.004, 1.546)	1.21	(0.974, 1.502)
Dual sensory loss	2.061^***^	(1.768, 2.404)	1.899^***^	(1.624, 2.220)	1.846^***^	(1.577, 2.161)	1.801^***^	(1.538, 2.110)
	**Baseline sensory loss and incidence of falls over 7 years**							
**Variables**	**Model 1**		**Model 2**		**Model 3**		**Model 4**	
	**OR**	**95%CI**	**OR**	**95%CI**	**OR**	**95%CI**	**OR**	**95%CI**
**No sensory loss**	**Reference**		**Reference**		**Reference**		**Reference**	
Hearing loss	1.345^***^	(1.161, 1.558)	1.299^***^	(1.119, 1.508)	1.295^***^	(1.115, 1.504)	1.282^**^	(1.104, 1.490)
Vision loss	1.174^*^	(0.998, 1.381)	1.103	(0.936, 1.299)	1.105	(0.938, 1.303)	1.089	(0.924, 1.285)
Dual sensory loss	1.623^***^	(1.442, 1.826)	1.487^***^	(1.318, 1.677)	1.477^***^	(1.307, 1.668)	1.454^***^	(1.287, 1.643)

*The results of the logistic regression models were expressed as odds ratios (OR) and 95% confidence intervals (CI). The analytic sample size was 7,623*.

*Model 1: unadjusted*.

*Model 2: adjusted for demographic factors including age and sex*.

*Model 3: adjusted for factors in Model 2, as well as social-economic and life style factors including BMI, marital status, educational level, smoking, alcohol consumption and physical activity status*.

*Model 4: adjusted for factors in Model 3, as well as physical disorders including hypertension, diabetes, dyslipidemia, kidney diseases, emotional disorders, memory-related diseases and stroke*.

* *p < 0.05*;

** *p < 0.01*;

*** *p < 0.001*.

In Table 4, we noticed that, compared to baseline sensory loss only, all types of persistent sensory loss statuses (HL/VL/DSL) were significantly and more strongly correlated with incidence of falls over 7 year of follow-up even after adjusting for multi-confounding factors. Mixed logistic regression showed that time-varying HL (OR: 1.203, 95% CI: 1.070–1.354) and DSL (OR: 1.479, 95% CI: 1.343–1.629) were significantly correlated with incident falls during longitudinal observation after adjusting multiple confounding factors. But it was not the case of VL.

**Table 4:** The impact of persistent/time-varying sensory loss status on incidence of falls over 7 years.

	**Persistent sensory loss and incidence of falls over 7 years**							
**Variables**	**Model 1**		**Model 2**		**Model 3**		**Model 4**	
	**OR**	**95%CI**	**OR**	**95%CI**	**OR**	**95%CI**	**OR**	**95%CI**
**No sensory loss**	**Reference**		**Reference**		**Reference**		**Reference**	
Hearing loss	3.379^***^	(1.808, 6.317)	3.235^***^	(1.720, 6.084)	3.302^***^	(1.745, 6.248)	3.282^***^	(1.728, 6.234)
Vision loss	2.599^*^	(1.102, 6.133)	2.431^*^	(1.023, 5.778)	2.382^*^	(0.992, 5.716)	2.596^*^	(1.076, 6.262)
Dual sensory loss	3.486^***^	(2.641, 4.603)	3.108^***^	(2.339, 4.130)	3.122^***^	(2.317, 4.207)	3.045^***^	(2.252, 4.116)
	**Time-varying sensory loss and incidence of falls over 7 years**							
**Variables**	**Model 1**		**Model 2**		**Model 3**		**Model 4**	
	**OR**	**95%CI**	**OR**	**95%CI**	**OR**	**95%CI**	**OR**	**95%CI**
**No sensory loss**	**Reference**		**Reference**		**Reference**		**Reference**	
Hearing loss	1.196^**^	(1.065, 1.343)	1.204^**^	(1.071, 1.354)	1.209^**^	(1.075, 1.360)	1.203^**^	(1.070, 1.354)
Vision loss	1.111	(0.969, 1.273)	1.102	(0.961, 1.265)	1.112	(0.969, 1.277)	1.11	(0.967, 1.274)
Dual sensory loss	1.504^***^	(1.366, 1.655)	1.489^***^	(1.353, 1.640)	1.491^***^	(1.354, 1.642)	1.479^***^	(1.343, 1.629)

*There were 1,220 participants whose sensory loss status was consistent over 7 years. The analytic sample size was 7,623 in the mixed logistic regression models for association between time-varying sensory loss and incidence of falls*.

*Model 1: unadjusted*.

*Model 2: adjusted for demographic factors including age and sex*.

*Model 3: adjusted for factors in Model 2, as well as social-economic and life style factors including BMI, marital status, educational level, smoking, alcohol consumption and physical activity status*.

*Model 4: adjusted for factors in Model 3, as well as physical disorders including hypertension, diabetes, dyslipidemia, kidney diseases, emotional disorders, memory-related diseases and stroke*.

* *p < 0.05*;

** *p < 0.01*;

*** *p < 0.001*.

## Discussion

This study contributes to the current literature examining the relationship between sensory loss and falls in Chinese population. We performed cross-sectional study and 7 year follow-up longitudinal observation to verify sensory loss including vision loss, hearing loss and dual sensory loss as risk factors of falls among older Chinese population for the first time.

The overall prevalence of falls in our sample was around 17.85%. such prevalence was similar to that found in other studies performed in other Asian community-dwelling older (5, 29–31). Since falls has become one of the most common causes of injuries among older people, which could lead to long-term disability or even death, exploration of risk factors associated with falls in older people was warranted. Among various potential risk factors of falls, sensory loss including vision loss and hearing loss has raised great concern in recent years.

### Single Vision Loss

A decrease in visual acuity could probably lead to inaccurate assessment of environmental obstacles and deficits in daily activities, which eventually prevent older people from avoiding falls and fall-related injuries (5). In our cross-sectional study, we found significant correlation between VL and prevalence of falls in our sample according to univariate logistic regression (OR: 1.330, 95% CI: 1.075–1.646). After adjusting various confounders including age, sex, BMI, marital status, educational level, smoking status, drinking status and physical activity, single VL was still significantly correlated to fall. To our surprise, with physical disorders added into the model, such correlation became less significant (*p* = 0.08, Model 4, Table 3).

Decline in vision may also contribute to the development of fear of falling, which are related to increased fall risk in older adults (32). However, we found relatively less significant correlation between baseline single VL with incidence of falls over 7 year of follow-up in our participants (Table 3). Such finding indicated that baseline single VL may not be an appropriate indicator for higher incidence of falls. Similar results were also found that time-varying VL was not significantly correlated with incidence of falls during longitudinal observation (Table 4). This may be explained by the fact that amelioration of poor vision status is relatively accessible and effective for older people in our country. For example, patients who had cataract at baseline and later underwent successful cataract surgery for vision improvement could report different vision status in the following surveys. Therefore, to persevere same exposure status, we then filtered our participants according to the criteria of consistent VL status to further verify the impact of persistent VL on incidence of falls over 7 years. Persistent single VL was found to be strongly and significantly correlated with incidence of falls over 7 year of follow-up, even after being adjusted for multi-confounding factors in all models (Table 4). These findings indicated that baseline VL may not be appropriate for prediction of higher incidence of falls in older Chinese population, but the persistence of poor vision status could probably lead to more falls in older Chinese. Alteration or amelioration of poor vision status would possibly lower the appearance of falls in older adults.

### Single Hearing Loss

HL has also been regarded as a risk factor of falls. HL contributes to balance difficulties, greater stride length variability and poorer postural control related to fall occurrence in older people (33–35). Numerous studies carried out across various ethnics and population in different countries have reached a consistent result of the potential correlation between HL and falls (36–40). Some researchers have also suggested that HL could be a clinical indicator of increased fall risk (11, 12). However, some researchers did not find significant correlation between HL and falls (13). Potential reasons may lie in the variability in how HL and falls were assessed and cohort characteristics. Similarly, several cross-sectional studies performed in our population have yielded mixed results as well (6, 10, 15, 16).

Thus, the present study provided important evidence on the correlation between single HL and falls in our older population from a nation-wide level according to both cross-sectional and longitudinal analyses for the first time. Baseline HL, persistent HL status and longitudinal time-varying HL were all found to be significantly associated with prevalence and incidence of falls in our samples even after being adjusted for all other confounders (all models, Tables 3, 4). These findings further indicated that single HL could be regarded as a risk factor of falls in middle-aged and older Chinese population. Our findings are consistent to several population-based nation-wide surveys performed in other countries (11, 12, 36, 37, 40).

HL, HL-related falls and HL interventions among older adults in our country should arouse enough concern. Interventions like wearing hearing aids for improvement of hearing status has been proved to be very helpful to improve postural stability and offer a significant public-health benefit for avoiding falls, particularly in older people (41, 42). However, according to the previous research in over 15 million older Chinese people with HL from the China National Sample Survey on Disability, researchers pointed out that there is less uptake of hearing aid use than expected (43). Reasons for the poor uptake of hearing aids included financial constraints, unfamiliarity with hearing aids, difficulties in manipulating hearing aids, and traditional attitudes toward HL in older people as a normal part of aging life (43). Furthermore, besides amplifying desired sounds, hearing aids would amplify noises as well, thus making users feel too loud and noisy. Such muffled effect also jeopardize their belief in hearing aid (43). Thus, we need to realize that the hearing healthcare services for older people in China is still under-developed and worthy of further improvement in the future.

### Dual Sensory Loss

In old age, sensory impairments often coexist. Thus far, the combined effects of HL and VL on falls have been barely explored in our population. According to the present study, the DSL group had the highest prevalence of falls among these sensory loss groups (22.4%, *n* = 3,163). The correlation between DSL and falls was apparently stronger than that between single sensory loss and falls (DSL: OR: 2.061, 95% CI: 1.768–2.404; HL: OR: 1.503, 95% CI: 1.240–1.820; VL: OR: 1.330, 95% CI: 1.075–1.646). Baseline DSL, persistent DSL status and time-varying DSL were all found to be significantly associated with prevalence and incidence of falls in our samples in both cross-sectional and longitudinal analyses even after being adjusted with all other confounders (all models, Tables 3, 4). These consistent results in the present study indicated the strong correlation between DSL and falls in middle-aged and older Chinese people for the first time.

Some researchers have also noticed the relatively higher risk of combined effect of DSL on falls in older people, which is consistent to our results (44–46). Older people with DSL may be exposed to jointly negative influences of HL and VL. Concomitant dysfunction of both the cochlear and vestibular sense organs were ubiquitous in older people with HL (36). On the other hand, weakened vestibulo-ocular reflex and worse balance maintenance could also be found in older people with a decrease in visual acuity (5, 47). In addition, older people with DSL may develop a greater fear of falling behavior, reduced mobility, restricted activity and a decline in social interactions, which could further lead to sarcopenia, depression, poorer cognitive status, and reduced attentional resources. All these factors could contribute to the increased incidence of falls (17, 18, 32, 36, 46, 48).

### Strengths and Limitations

There are several strengths in our study. First, CHARLS is a national study with large sample size, and the national representativity of CHARLS has been widely recognized and acknowledged. Thus, our work could be generalized to the entire country. Second, to our knowledge, the current study is the first nation-wide Chinese population-based study to verify the sensory-fall association among middle-aged and older population according to both cross-sectional study and longitudinal observation over 7 years. Results in our study could not only be used as evidence for falls-prevention among older population in China but also reference for future studies in other countries (especially in developing countries). Lastly, multiple fall-associated factors were included and adjusted in this study analyses, which could otherwise potentially confound the relationship between sensory loss and falls.

Meanwhile, we acknowledge some limitations. First, data of sensory loss and falls was collected by self-reports. Date for falls and frequency of falls was unavailable as well. Although this method has been used in previous studies (49–53), possible misclassification of sensory loss status or inaccurate reports may lead to bias. Also, the causal effects of sensory loss on falls could not be reached according to the present study. Second, some previously reported confounding factors of incident falls, such as physical environment, sarcopenia and nutrient intake were not available in CHARLS and were not adjusted in our study. Third, in a longitudinal observation over 7 years in older population, it is inevitable that the attrition in the panel over time could not be completely random. Lost follow up in following visits and the exclusion criteria of the present study could probably lead to sampling bias as well. Whatsoever, CHARLS is the first nationally representative survey of the health status and well-being in middle-aged and older population in China, which provides high-quality data of massive amounts of personal health-related information.

## Conclusion

Our work is the first to verify sensory loss including vision loss, hearing loss and dual sensory loss as risk factors of falls among older Chinese population according to cross-sectional study and 7 year follow-up longitudinal observation. Hearing loss and dual sensory loss are significantly associated with both prevalence and increased incidence of falls over 7 year's observation in middle-aged and older Chinese population. Persist or altered vision loss status could exert divergent influences on incidence of falls. These findings deserve further consideration in the development of falls-prevention public health policies for older population in China.

## Data Availability Statement

The datasets presented in this study can be found in online repositories. The names of the repository/repositories and accession number(s) can be found at: The current study is a secondary analysis of public data of CHARLS. The original datasets of CHARLS is accessible on http://charls.pku.edu.cn/.

## Ethics Statement

The studies involving human participants were reviewed and approved by The Biomedical Ethics Review Committee of Peking University. The patients/participants provided their written informed consent to participate in this study. Written informed consent was obtained from the individual(s) for the publication of any potentially identifiable images or data included in this article.

## Author Contributions

MZ, YZ, HL, and XS designed the research. JL, YH, and YL analyzed the data. YZ drafted the manuscript. YZ and YH contributed equally to this research and should be considered as equivalent authors. All authors read and approved the final manuscript.

## Funding

This research was supported by the National Natural Science Foundation of China (81730026), National Key R&D Program (2017YFA0105301), Multi-center Clinical Research Project from Shanghai Jiao Tong University School of Medicine (DLY201813), Science and Technology Commission of Shanghai Municipality (19495800700), Shanghai Hospital Development Center (SHDC2020CR2040B), Shanghai Hospital Development Center (SHDC2020CR5014), Shanghai Natural Science Foundation (19ZR1440900), Shanghai Pujiang Program (2019PJD047), Project of Shanghai Putuo District People's Hospital (2021rmlcky01). All sponsors had no role in the study design, data collection, and analysis, decision to publish, or preparation of the manuscript.

## Conflict of Interest

The authors declare that the research was conducted in the absence of any commercial or financial relationships that could be construed as a potential conflict of interest.

## Publisher's Note

All claims expressed in this article are solely those of the authors and do not necessarily represent those of their affiliated organizations, or those of the publisher, the editors and the reviewers. Any product that may be evaluated in this article, or claim that may be made by its manufacturer, is not guaranteed or endorsed by the publisher.
